# Whole mastic resin ameliorates halitosis and gingivitis in dogs and cats infected with *Porphyromonas gulae*

**DOI:** 10.1038/s41598-025-27244-x

**Published:** 2025-12-08

**Authors:** Mao Kaneki, Nana Komai, Chiharu Ohira, Tomoki Fukuyama

**Affiliations:** 1 Laboratory of Veterinary Pharmacology, School of Veterinary Medicine, Azabu University, 1-17-71 Fuchinobe, Chuo-ku, Sagamihara-shi, Kanagawa, 252-5201 Japan; 2 Center for Human and Animal Symbiosis Science, Azabu University, 1-17-71 Fuchinobe, Chuo-ku, Sagamihara-shi, Kanagawa, 252-5201 Japan

**Keywords:** Mastic, Periodontal disease, *Porphyromonas gulae*, Dogs, Cats, Halitosis, Dental diseases, Inflammation

## Abstract

Mastic, a natural resin, has long been used to prevent periodontal disease, but most studies focus on mastic extracts rather than the resin itself. This study investigated the therapeutic potential of whole mastic resin against *Porphyromonas gulae*-associated halitosis and inflammatory responses in vitro and in vivo. Mastic (0.06%–1%) was evaluated for bactericidal, anti-halitosis, and anti-inflammatory effects using *P. gulae* and macrophage cell lines. Oral mastic gel (5%) was applied daily in dogs and cats with *P. gulae*-positive periodontal disease. Mastic reduced *P. gulae* viability in a dose-dependent manner and rapidly inhibited hydrogen sulfide and methyl mercaptan, the main halitosis factors, within five minutes. It also significantly suppressed pro-inflammatory cytokines (IL-1β, IL-6, TNF-α) and mitogen-activated protein kinase signaling. In clinical trials, daily mastic treatment for one month decreased halitosis, gingivitis, plaque accumulation, and *P. gulae* activity in both dogs and cats. The effect on plaque was more pronounced in cats, suggesting species-specific responses. No cytotoxicity was observed. These findings demonstrate that oral administration of whole mastic resin provides rapid antibacterial, anti-halitosis, and anti-inflammatory effects, supporting its potential as a therapeutic option for managing periodontal disease in companion animals.

**Supplementary Information:**

The online version contains supplementary material available at 10.1038/s41598-025-27244-x.

## Introduction

Mastic is a natural resin obtained from the stems and leaves of the *Pistacia lentiscus* tree, which is native to the Mediterranean Basin. However, commercial production of high-quality mastic resin occurs almost exclusively on the Greek island of Chios^1,2^. Chewing mastic has long been considered for the prevention of periodontal disease (PD) in European countries. Previous studies have demonstrated that the mastic extract significantly inhibits the growth of *Porphyromonas gingivalis*, a major periodontal pathogen; thus, it is considered an alternative antibacterial agent for preventing PD^3,4^. In addition to its antibacterial effects, several studies have indicated that mastic treatment has anti-inflammatory responses including the downregulation of interleukin (IL)-8, NF-κB p65, and tumor necrosis factor- α (TNF-α)-induced oxidative stress *in vitro*^2,5,6^. Our group has also demonstrated that topical treatment with mastic significantly ameliorates the inflammation and itching associated with allergic contact dermatitis and atopic dermatitis by modulating keratinocyte activation^7^. A significant downregulation of cytokine secretion by mastic cells was observed, particularly in keratinocytes. The novelty of our study lies in the use of mastic itself, not an extract. Most scientific studies and commercial products focus on mastic extracts or essential oils rather than the whole resin itself^8–10^. The effects of pure mastic resin remain largely unexplored, despite historical and anecdotal evidence suggesting its efficacy in oral health and PD prevention^7,11^. The objective of this study was to confirm the effectiveness of mastic against periodontal pathogens and the related halitosis and inflammation in vitro and in vivo.

The progression of PD and tooth loss are associated with aging in both humans and dogs^12,13^. The prevalence of PD in dogs is rapidly increasing, with PD-associated bacterial infections found in over 80% of adult dogs^14^. As PD becomes chronic, inflammation gradually spreads to periodontal tissues, forming irreversible deep periodontal pockets in the gingival sulcus^15^. Recent evidence indicates that chronic periodontitis is associated with systemic diseases^16^. However, preventive and therapeutic options for dogs and cats remain limited, and there is little research on the efficacy of whole mastic resin, although mastic extracts have shown beneficial effects. To investigate the effects of mastic on PD in companion animals, *Porphyromonas gulae* (*P. gulae*) was selected as the representative periodontal pathogen because it is the predominant species associated with periodontal disease in dogs and cats. Several studies have demonstrated that *P. gulae* is frequently isolated from subgingival plaque in dogs with PD, showing strong pathogenic similarities to *P. gingivalis* in humans and playing a central role in disease progression^17,18^. To address this gap, the present study evaluated the bactericidal, anti-halitosis, and anti-inflammatory effects of whole mastic resin in vitro using bacterial and macrophage cell models, and validated these findings in vivo through clinical trials in dogs and cats with naturally occurring PD. We hypothesize that whole mastic resin can directly inhibit *P. gulae* growth, suppress halitosis-related gas production, and reduce pro-inflammatory cytokine secretion, thereby improving periodontal health. The primary research question is: *Does daily oral administration of whole mastic resin effectively manage P. gulae–associated PD and halitosis in dogs and cats?*

## Results

### Bactericidal effects of mastic on P. gulae

**Fig. 1:**
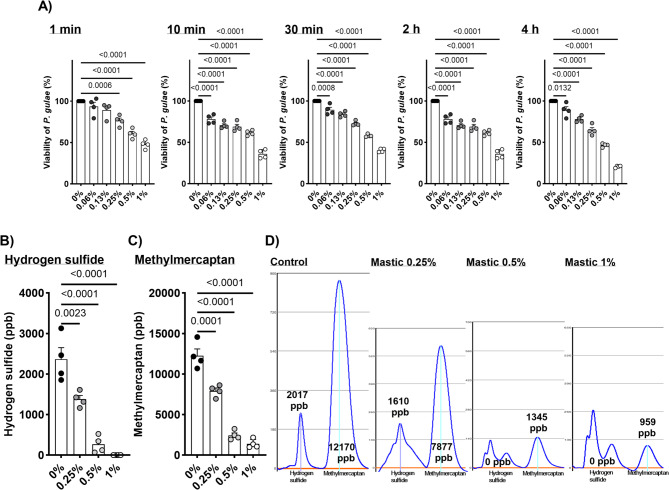
The direct influence of mastic on *P. gulae* activities. (**A**) Bactericidal effects of mastic against *P. gulae* at each time point. (**B**) Hydrogen sulfide and (**C**) methyl mercaptan production by *P. gulae* following 5 min co-incubation with mastic. (**D**) Representative images of gas chromatography of hydrogen sulfide and methyl mercaptan. Each result is presented as the mean (% or ppb) ± 1 SEM. n = 4 per group. p < 0.05 (Dunnett’s multiple comparison test) vs. control (0%) group. *P. gulae*, *Porphiromonas gulae*.

The bactericidal effects of mastic on *P. gulae* are shown in Fig. 1A. Treatment with mastic at concentrations higher than 0.25% significantly inhibited *P. gulae* viability even after 1 min of exposure compared to the vehicle control (0%). At extended incubation times, the bactericidal effect became more pronounced in a concentration-dependent manner. For example, 0.5% mastic treatment reduced bacterial viability to approximately 50% at 4 h, while 1% mastic treatment reduced viability to 50% at 10 min, 40% at 30 min, 30% at 2 h, and 20% at 4 h. Even low concentrations (0.06% and 0.13%) exhibited significant bactericidal activity, but longer exposure times (≥ 10 min) were required to achieve notable inhibition.

### Inhibitory effects of mastic on hydrogen sulfide and methyl mercaptan generation by P. gulae

Halitosis often associated with PD in dogs and cats, is primarily caused by *P. gulae-*generated hydrogen sulfide and methyl mercaptan^19,20^. The inhibitory effects of mastic on the production of these compounds by *P. gulae* were analyzed in this study (Fig. 1B-D). Short-term (5 min) treatment with mastic (0.25%, 0.5%, and 1%) significantly reduced hydrogen sulfide and methyl mercaptan production in a dose-dependent manner compared with the vehicle control treatment (0%). The direct deodorant property of the mastic was confirmed using a methyl mercaptan standard, with no observable influence from mastic co-incubation (Supplemental Fig. 1).

### Inhibitory effects of mastic on pro-inflammatory cytokines secretion in murine macrophages induced by P. gulae- or LPS

**Fig. 2:**
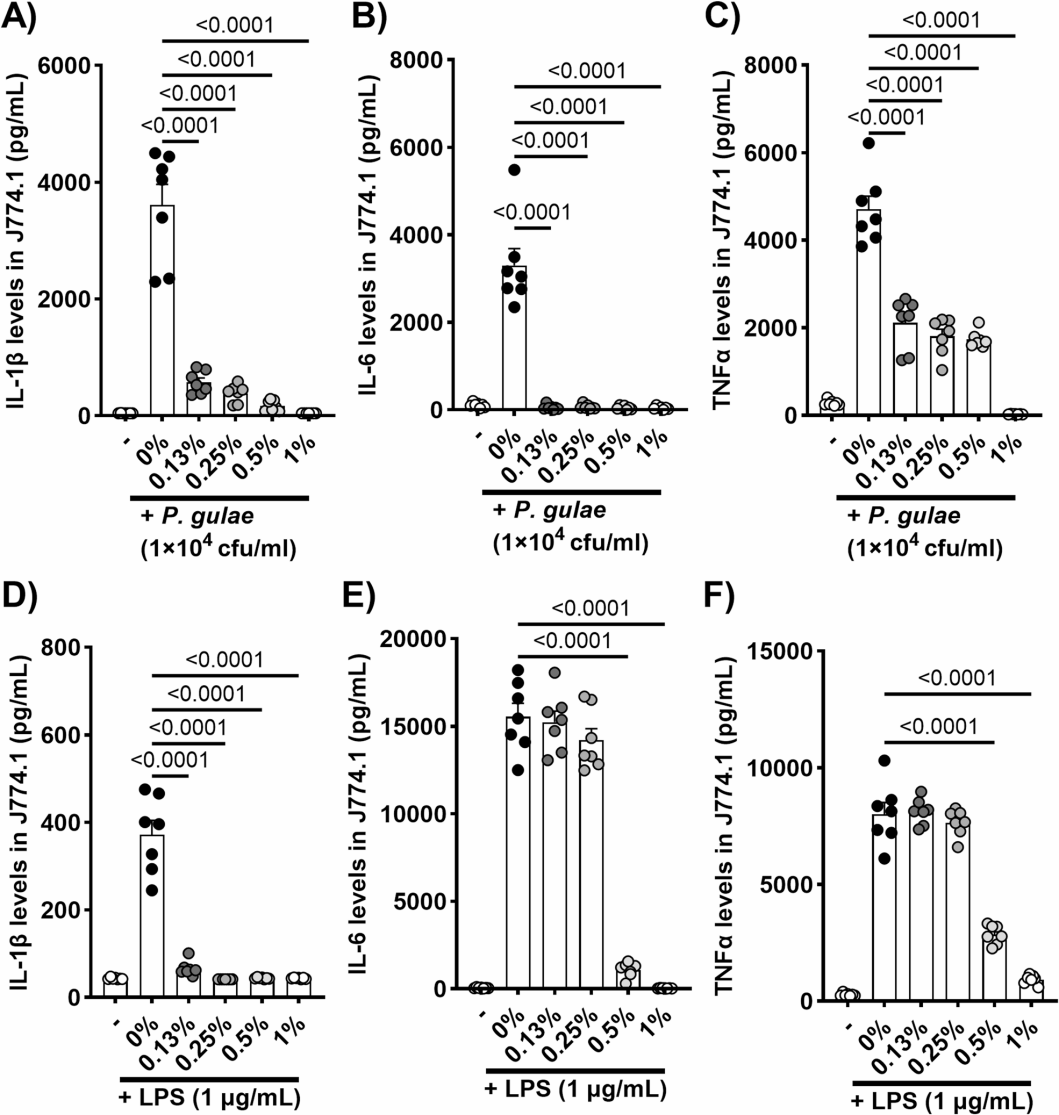
Inhibitory effects of mastic on the production of pro-inflammatory cytokines by murine macrophage cell line (J774.1). (**A**) IL-1β, (**B**) IL-6, and (**C**) TNF-α secretion by the *P. gulae*-infected murine macrophage. (**D**) IL-1β, (**E**) IL-6, and (**F**) TNF-α secretion by the LPS-induced murine macrophage. Each result is presented as the mean (pg/mL) ± 1 SEM. n = 7 per group. p < 0.05 (Dunnett’s multiple comparison test) vs. *P. gulae*-infected control (-) group. IL, interleukin; LPS, lipopolysaccharide; TNF, tumor necrosis factor.

The anti-inflammatory properties of mastic against PD symptoms were evaluated by examining *P. gulae*- and LPS-induced cytokine production in a murine macrophage cell line (J774.1). The influence of mastic treatment on *P. gulae*-induced IL-1β, IL-6, and TNFα secretion are shown in Fig. 2A–C. Mastic at various concentrations (0.13%, 0.25%, 0.5%, and 1%) significantly suppressed cytokine production in a dose-dependent manner, with notable effects at the lowest concentration (0.13%). Interestingly, a similar inhibitory pattern was observed in LPS-induced IL-1β, IL-6, and TNF-α secretion (Fig. 2D–F); however, low concentrations of mastic (0.13% and 0.25%) significantly reduced only IL-1β secretion. Importantly, no cytotoxicity was observed at any concentration of mastic tested, as confirmed by LDH assay (data not shown).

### Inhibitory effects of mastic on P. gulae-induced phosphorylation of mitogen-activated protein kinase (MAPK) in J774.1

**Fig. 3:**
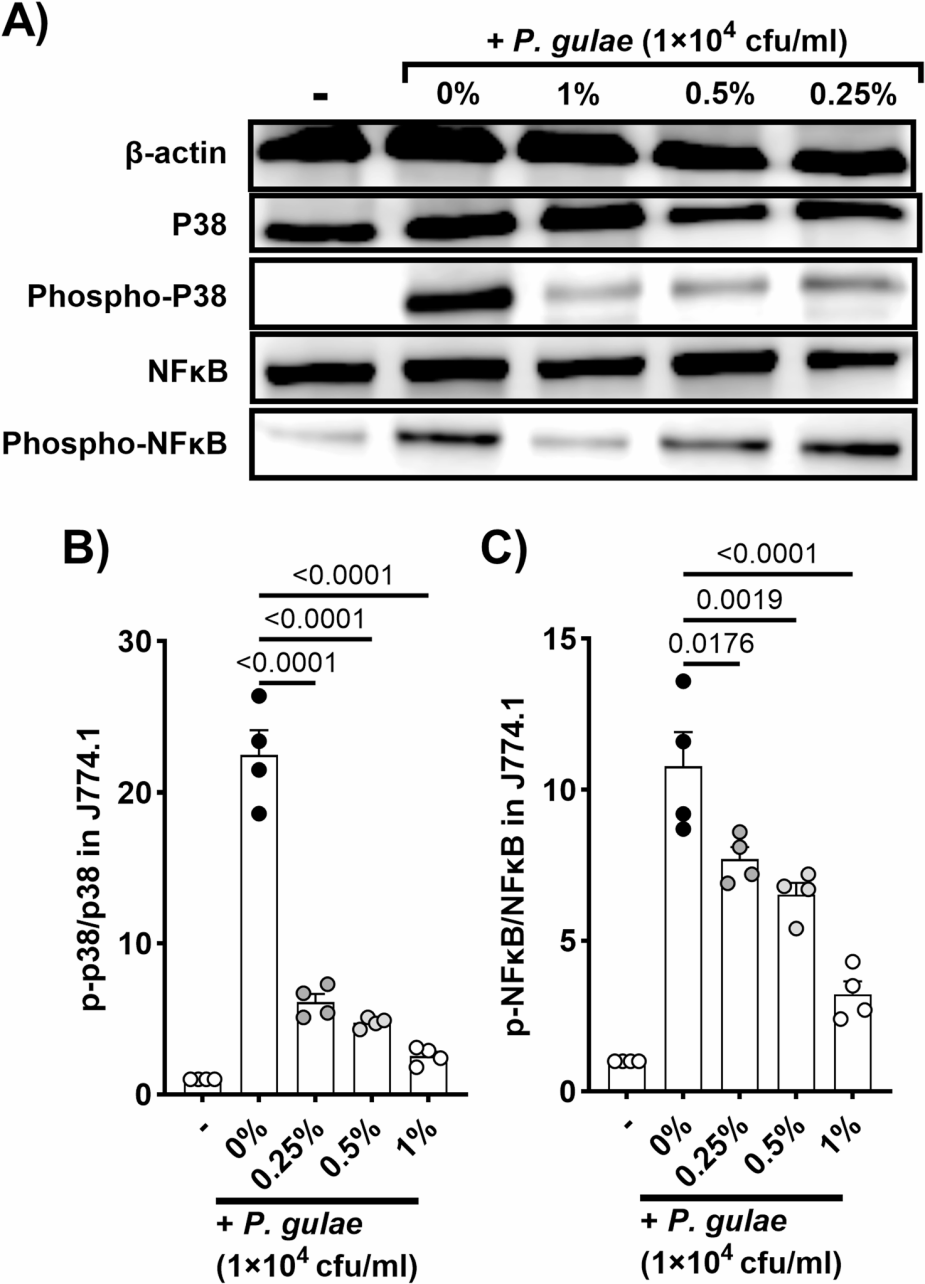
Impact of mastic on phosphorylation of MAPKs induced by *P. gulae* in murine macrophage cell line (J774.1). (**A**) Representative images of western blot analysis. The grouping of gels/blots cropped from different parts of the same gel and exposures were made explicit. Mastic treatment significantly reduced the phosphorylation of (**B**) p38 and (**C**) NFκB induced by *P. gulae* infection in a dose-dependent manner. Each result is presented as the mean ± standard error of the mean (SEM). N = 4 per group. p < 0.05 (Dunnett’s multiple comparison test) vs. control group. MAPK, mitogen-activated protein kinase.

The secretion of pro-inflammatory cytokines is regulated by MAPK pathway which includes p38 and NFκB. The effects of mastic on *P. gulae*-induced phosphorylation of p38 and NFκB were assessed in J774.1 cells. Mastic treatment at concentrations of 0.25%, 0.5%, and 1% significantly inhibited *P. gulae*-induced phosphorylation of p38 and NFκB in a dose-dependent manner (Fig. 3A–C).

### Inhibitory effects of mastic on the secretion of pro-inflammatory cytokines by P. gulae-induced canine macrophages

**Fig. 4:**
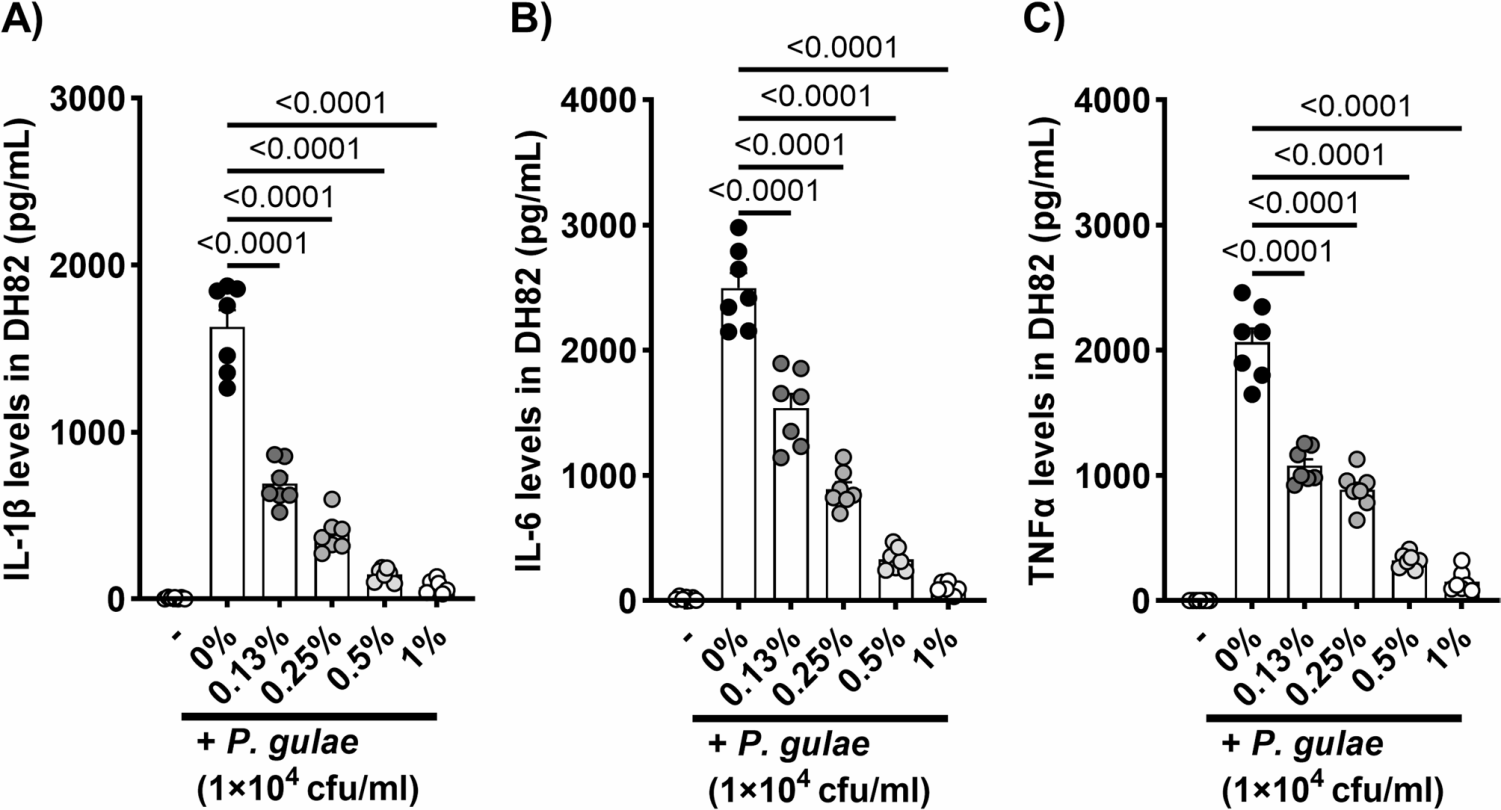
Inhibitory effects of mastic on the production of pro-inflammatory cytokines by canine macrophage cell line (DH82). (**A**) IL-1β, (**B**) IL-6, and (**C**) TNF-α secretion by the *P. gulae*-infected canine macrophage. Each result is presented as the mean (pg/mL) ± 1 SEM. n = 7 per group. p < 0.05 (Dunnett’s multiple comparison test) vs. *P. gulae*-infected control (-) group. IL, interleukin; TNF, tumor necrosis factor.

To evaluate the efficacy of mastic in dogs, its inhibitory effects on cytokine secretion were assessed in canine macrophage cells (DH82). Treatment with mastic significantly reduced *P. gulae*-induced IL-1β, IL-6, and TNF-α secretion at concentrations of 0.13%, 0.25%, 0.5%, and 1%, compared to the vehicle control group, in a dose-dependent manner (Fig. 4A–C).

### Daily oral treatments of mastic in dogs and cats with P. gulae-positive PD

**Fig. 5:**
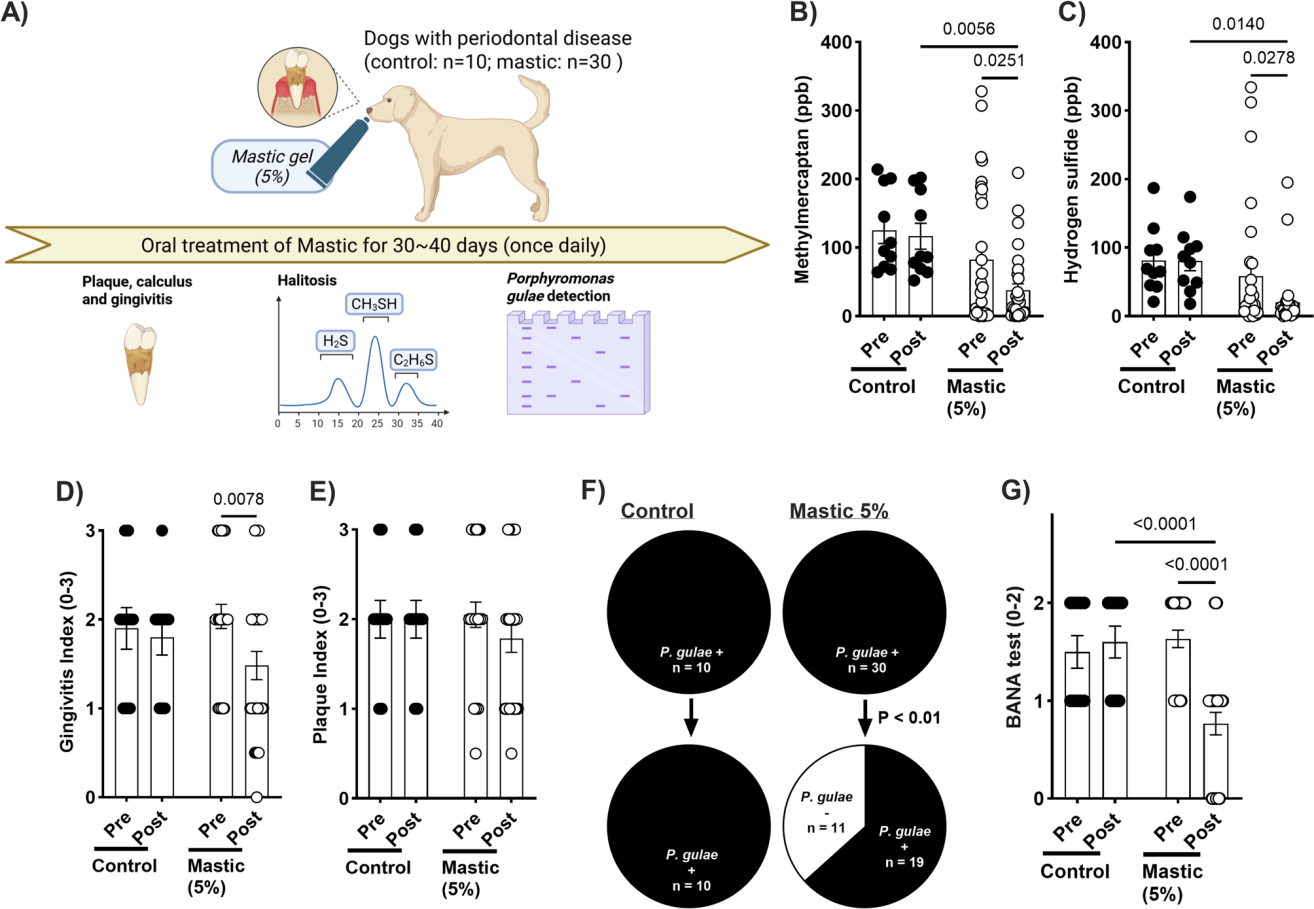
Changes in periodontal conditions in dogs with *P. gulae* before and after daily mastic (5%) gel treatment. (**A**) Experimental schedule. (**B**) Methyl mercaptan (ppb) and (**C**) hydrogen sulfide (ppb) levels in expired breath were significantly reduced after 30–40 days of mastic treatment. (**D**) Gingivitis scores were significantly improved compared to pre-treatment values. (**E**) Plaque index showed no significant change following mastic treatment. (**F**) PCR analysis revealed a significant reduction in *P. gulae* levels after treatment, with no change in the vehicle control group. (**G**) Hydrolysis of BANA, indicative of periodontal pathogen activity, was suppressed by mastic treatment compared to pre-treatment and vehicle control levels. Data are presented as mean ± 1 SEM; n = 30 for mastic, n = 10 for vehicle control; p < 0.05 (uncorrected Fisher’s LSD test). BANA, N-benzoyl-DL-arginine-2-naphthylamide; PCR, polymerase chain reaction.

**Fig. 6:**
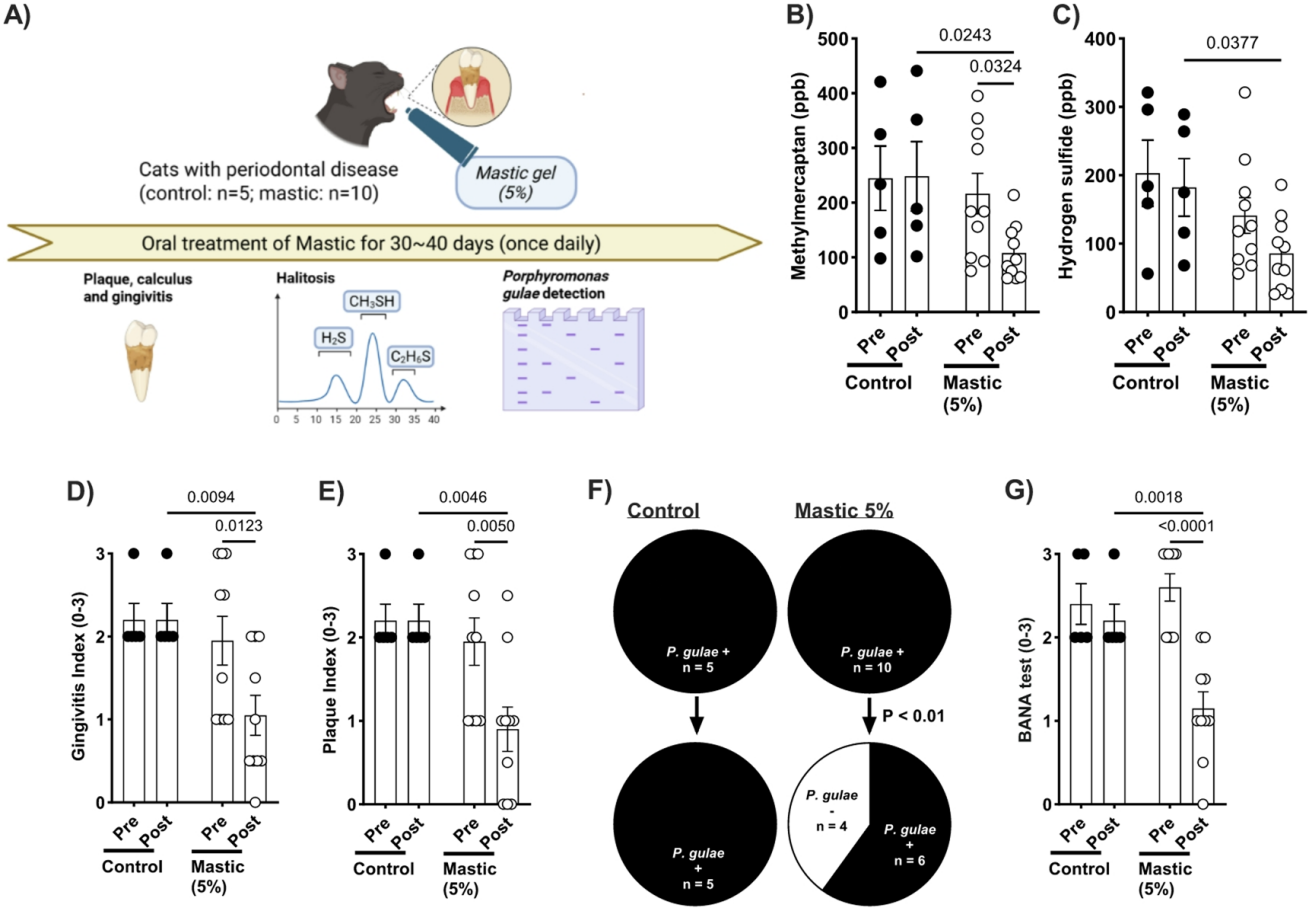
Changes in periodontal conditions in cats with *P. gulae* before and after daily mastic (5%) gel treatment. (**A**) Experimental schedule. (**B**) Methyl mercaptan (ppb) and (**C**) hydrogen sulfide (ppb) levels in expired breath were significantly reduced after 30–40 days of treatment. (**D**) Gingivitis and (**E**) plaque index were significantly improved compared to pre-treatment and vehicle control values. (**F**) DNA detection of *P. gulae* and (**G**) BANA hydrolysis were significantly suppressed by mastic treatment compared to pre-treatment and vehicle control levels. Data are presented as mean ± 1 SEM; n = 10 for mastic, n = 5 for vehicle control; p < 0.05 (uncorrected Fisher’s LSD test). BANA, N-benzoyl-DL-arginine-2-naphthylamide; PCR, polymerase chain reaction.

The bactericidal, anti-halitosis and anti-inflammatory effects of mastic in vitro were validated through in vivo clinical trials in dogs and cats. In dogs with PD, daily oral treatment with 5% mastic gel significantly decreased methyl mercaptan and hydrogen sulfide levels in expired breath (Fig. 5B, C). Sensory evaluation of halitosis by pet owners supported the gas chromatography findings (Supplemental Fig. 2). Significant inhibition of gingivitis was observed after one month of treatment with 5% mastic, whereas there was no significant impact on the plaque index (Fig. 5D, E). DNA detection and activity of *P. gulae* were significantly inhibited by the 5% mastic treatment compared to the vehicle control treatment and pre-treatment levels (Fig. 5F, G). A similar efficacy of mastic treatment was observed in cats with PD. The methyl mercaptan and hydrogen sulfide levels in the expired breath were significantly decreased by the mastic treatment compared to the vehicle treatment (Fig. 6B, C), which was corroborated by the significant decrease in halitosis in the pet owners’ sensory evaluation (Supplemental Fig. 3). Interestingly, the effects of mastic treatment on gingivitis and the plaque index were more pronounced in cats than in dogs. Significant improvements were observed in both gingivitis and plaque index compared to the vehicle control treatment and pre-treatment levels (Fig. 6D, E). Furthermore, DNA detection and activity of *P. gulae* were significantly reduced by 5% mastic treatment compared to that of the vehicle control treatment and pre-treatment levels (Fig. 6F, G).

A summary of key pre- and post-treatment outcomes for gingivitis, plaque, halitosis-related gases, *P. gulae* load, BANA activity, and cytokine levels is presented in Table 1, highlighting the overall effects of daily 5% mastic gel treatment in dogs and cats.

**Table 1:** Key pre- and post-treatment outcomes following daily 5% mastic gel in dogs and cats with *P. gulae*-associated periodontal disease.

Outcome	Dogs (n = 30)	Cats (n = 10)	Vehicle control
Gingivitis score	↓ significant	↓ significant	NS
Plaque index	NS	↓ significant	NS
Methyl mercaptan	↓ significant	↓ significant	NS
Hydrogen sulfide	↓ significant	↓ significant	NS
*P. gulae* DNA	↓ significant	↓ significant	NS
BANA activity	↓ significant	↓ significant	NS
Cytokines	↓ significant (canine/macrophage)	–	–

## Discussion

PD begins with gingivitis, where periodontopathic bacteria form a biofilm in the gingival sulcus between the teeth and gingiva, causing redness and swelling of the gingival margin^15^. As the disease progresses, healthy periodontal tissue is lost, resulting in tooth mobility and eventual tooth loss^21^. The situation in dogs and cats is much more severe than in humans, as daily dental care depends on the pet owner, and professional dental procedures require general anesthesia, even for elderly animals. Improvements in living conditions and advancements in veterinary medicine have extended the life-span of dogs and cats; however, the progression of PD has rapidly accelerated, especially in animals over 10 years of age^22^. An epidemiological study by Shirahata, et al.^12^ indicated a significant difference in the number of residual teeth between *P. gulae-*negative and *P. gulae*-positive dogs older than 100 months. Yasuda, et al.^14^ also suggested that PD in small-breed dogs gradually progresses from birth with chronic PD developing within several years. Therefore, early preventive dental care is critical, especially for small-breed dogs, to prevent the establishment of oral infections with highly pathogenic bacteria, such as *P. gulae*. In this study, we focused on mastic and examined its bactericidal, anti-halitosis, and anti-inflammatory potential through in vitro and clinical studies in dogs and cats.

To investigate the effects of mastic on PD in companion animals, *P. gulae* was used as a representative periodontal pathogen in dogs and cats in this study. *P. gulae* has been isolated from oral swabs of dogs with PD^23^. In humans, red complex bacteria, such as *P. gingivalis*, *T. denticola*, and *Tannerella forsythia* are dominant in the development of PD. However, in dogs, broad-range polymerase chain reaction and sequencing methods have revealed that approximately 70% of periodontal pathogens are *P. gulae*, whereas the frequency of *P. gingivalis* is extremely low^23^. Our recent epidemiological studies also indicated a strong correlation between *P. gulae* infection and the development of PD in dogs^12,14^. In this study, we first identified the direct effects of mastic on cell viability and the generation of hydrogen sulfide and methyl mercaptan in vitro. The viability of *P. gulae* was significantly decreased by mastic treatment in a dose-dependent manner. When compared with our previous studies, the bactericidal property of mastic against *P. gulae* was much higher than that of folic acid or catechin^19,20^. Most importantly, studies reported that mastic exhibited a bactericidal effect as early as one minute after treatment, a result not observed with folic acid or catechin^19,20^. For dental care applications in dogs and cats, the maximum retention time of mastic in the oral cavity was approximately 30 min, based on our previous study and preliminary pilot observations of mastic gel persistence, which were confirmed through visual inspection by veterinarians and owners’ reports of gel dissolution^13^. The immediate effect of mastic on *P. gulae* is a significant advantage when considering its in vivo efficacy. Similar inhibitory effects of mastic on the generation of hydrogen sulfide and methyl mercaptan, which are the major sources of halitosis, were observed in vitro. While halitosis is not directly associated with the pathogenesis of PD, it is an important factor in fostering better personal relationships^24^. Indoor breeding of dogs and cats has become more common, especially in urban areas, owing to housing constraints and associated costs^25^. Severe halitosis in household dogs and cats can negatively impact the bond between pets and their owners^26^. Therefore, improving PD and associated halitosis benefits not only the health aspects of the animals but also the living environment. The rapid and potent effects of mastic (within five minutes) on *P. gulae-*associated halitosis in vitro represent an added advantage for its in vivo application in dogs and cats. However, we acknowledge that although significant reductions in halitosis-related gases were observed as early as 5 min after treatment, this immediate effect may not reflect sustained odor control. Further studies are needed to evaluate the persistence of halitosis reduction over time.

Gingival inflammation induced by periodontal pathogens and their lipopolysaccharides (LPS) can progress from reversible gingivitis to irreversible periodontitis characterized by bone lysis^27^. Tissue regeneration and implant techniques are rapidly advancing in human medicine, however, these approaches are not realistic in veterinary medicine because of the anatomical differences between dog breeds^28,29^. Therefore, early-stage care aimed at preventing inflammation is important to preserve teeth later in life. In this study, we examined the inhibitory effects of mastic on pro-inflammatory cytokine production and related MAPK signaling. Macrophages play a key role in the development of PD by detecting periodontal pathogens and initiating inflammatory response through cytokine production^30^. Initially, we analyzed the prevention of *P. gulae*-induced IL-1β, IL-6, and TNF-α production through co-treatment with mastic in murine and canine macrophages. Significant reductions in cytokine levels were observed at non-cytotoxic mastic concentrations. Additionally, the significant inhibition of phosphorylation of p38 and NFκB, components of the MAPK pathway associated with pro-inflammatory cytokine production, by mastic co-treatment, corroborates the observed cytokine suppression^31,32^. While the downregulation of these cytokines is partially attributable to the bactericidal properties of mastic, cytokine production induced by LPS was also significantly suppressed by mastic co-treatment. Our previous study on topical mastic treatment in allergic and atopic dermatitis mouse models showed strong anti-inflammatory properties independent of its bactericidal effects^7^. Thus the anti-inflammatory effects of mastic in PD are likely a combination of dependent and independent bactericidal effects.

The in vitro effects of mastic were validated through in vivo clinical trials in dogs and cats with PD. Once-daily treatment with 5% mastic gel for 30–40 days significantly ameliorated methyl mercaptan and hydrogen sulfide levels compared to the control and pre-treatment values. Quantitative evaluations of halitosis closely correlated with the sensory evaluations of pet owners in both dogs and cats. This correlation highlights the importance of pet owner satisfaction in maintaining motivation for daily care routines. The gingivitis index, which reflects PD-related inflammation, was also significantly improved by mastic treatment compared to the pre-treatment values in both dogs and cats. This improvement is likely accompanied by the inhibition of pro-inflammatory cytokine secretion by macrophages, as observed in in vitro experiments. While the plaque index showed no significant changes following mastic treatment in dogs, it improved significantly in cats. The bacterial effect of mastic may have influenced the plaque index; however, the impact was limited over the short treatment period. Moreover, maintaining oral hygiene in cats is particularly challenging, as daily home care is often impractical for owners. This may contribute to delayed diagnosis or less frequent treatment compared with dog^33^. The better plaque index results in cats compared to dogs in our study may reflect species-specific differences in oral microbiota composition, plaque accumulation dynamics, or owner compliance at baseline. Moreover, DNA detection of *P. gulae* and the secretion of BANA were significantly decreased by mastic treatment, corroborating the in vitro bactericidal effects of mastic. As summarized in Table 1, mastic treatment consistently reduced gingival inflammation, halitosis, and bacterial activity across both species, although plaque reduction was minimal in dogs.

According to our results, the therapeutic effects of whole mastic resin may result from a combination of direct bactericidal activity and modulation of host inflammatory responses. Mastic contains bioactive compounds such as triterpenes and essential oils, which may disrupt bacterial cell membranes, inhibit metabolic enzymes, and reduce the production of halitosis-related gases. In addition, these compounds appear to interfere with MAPK signaling pathways in macrophages, suppressing pro-inflammatory cytokine secretion. Thus, mastic’s effects are likely mediated by both bacteria-targeted and host-modulatory mechanisms, contributing to the observed improvements in periodontal health and halitosis.

On the other hand, this study has several limitations. First, the clinical evaluation was limited to a short-term treatment period (30–40 days), and the long-term durability of mastic’s effects on periodontal health and halitosis remains unknown. Second, only dogs and cats were included, with a relatively small sample size in cats, limiting generalizability to other companion animals. Third, while significant improvements in gingivitis and halitosis were observed, plaque reduction was minimal in dogs, suggesting that mastic alone may not fully address all aspects of periodontal disease and that combination with mechanical cleaning may be required. Additionally, in vitro experiments focused on planktonic *P. gulae*, whereas periodontal pathogens predominantly exist within biofilms; future studies are needed to evaluate mastic’s efficacy against biofilm-associated bacteria. Finally, cytokine assays were performed in murine and canine macrophages, and feline immune responses were not directly assessed due to the lack of commercially available cell lines. Murine J774.1 cells were used as a standard macrophage model with well-characterized signaling pathways, enabling comparison with existing literature. Canine DH82 cells were included to reflect veterinary relevance. Feline macrophage cell lines are not commercially available and primary feline macrophages are difficult to obtain, which limited their inclusion in this study. Future studies should investigate long-term efficacy, lower mastic concentrations, biofilm-targeted effects, and broader species inclusion to fully characterize the therapeutic potential of whole mastic resin.

In summary, mastic treatment exhibited strong bactericidal, anti-halitosis, and anti-inflammatory effects against *P. gulae-*associated PD in vitro and in vivo. The anti-halitosis and anti-inflammatory effects of mastics are attributable to their potent bactericidal effects. However, this study has limitations. There was only a slight reduction in existing plaque. Therefore, daily and consistent treatment should be mandatory to achieve sufficient therapeutic effects. Additionally, the therapeutic effects were evaluated only at a high concentration (5%), underscoring the need for future studies to evaluate the efficacy of lower concentrations.

## Methods

### Preparation of the mastic

Chios mastic resin was provided by Sosin Co., Ltd. (Tokyo, Japan). For in vitro experiments, highly purified mastic was dissolved directly in the corresponding cell culture medium to ensure solubility and compatibility with bacterial or cell culture conditions, and then adjusted to final concentrations of 0.06–1%. For in vivo clinical trials, the mastic was dissolved in glycerin and cellulose gum (Sosin Co., Ltd.) to a final concentration of 5%. The vehicle gel, composed of glycerin and cellulose gum, was selected to match the physical properties of the mastic gel without containing active mastic resin, ensuring that any observed effects could be attributed to the resin itself rather than the gel base.

### Bactericidal effects of mastic on P. gulae

*P. gulae* strain ATCC 51,700 (fimA type A) was obtained from the Japan Collection of Microorganisms (RIKEN BioResource Research Center, Ibaraki, Japan). *P. gulae* was cultured anaerobically at 37 °C for 72 h in BD BBL™ CDC Anaerobic 5% Sheep Blood Agar (Becton, Dickinson and Company, NJ, USA) and horse red cell contained Brucella broth (KYOKUTO PHARMACEUTICAL INDUSTRIAL CO., LTD., Tokyo, Japan). The bactericidal effect of mastic on *P. gulae* was assessed using the BacTiter-Glo™ Microbial Cell Viability Assay (Promega KK., Tokyo, Japan), which quantifies viable bacterial cells based on ATP measurement. Following co-incubation of *P. gulae* suspensions with various concentrations of mastic (0.06%, 0.13%, 0.25%, 0.5%, and 1%) for 1 min, 10 min, 30 min, 2 h and 4 h, bacterial viability was determined by measuring luminescence using a GloMax®-Multi Detection System (Promega KK.). The emitted luminescence is directly proportional to intracellular ATP levels, allowing quantitative evaluation of viable bacterial cells. Decreased luminescence relative to untreated controls was interpreted as a bactericidal effect. Bacterial inocula were adjusted to an optical density at 600 nm (OD₆₀₀) corresponding to approximately 1 × 10⁸ CFU/mL, which reflects clinically relevant bacterial loads reported in subgingival plaque of dogs with moderate-to-severe PD^18,34^. Inocula were prepared in the same manner for all experiments, and equivalent OD₆₀₀ values were used to ensure comparability across replicates. This concentration was selected to reflect clinically relevant bacterial loads reported in subgingival plaque. Bacterial suspensions of *P. gulae* (1 × 10⁸ CFU/mL) were mixed with Brucella broth containing mastic (0.06–1%) and incubated for 1 min, 10 min, 30 min, 1 h, 2 h, or 4 h. Negative controls consisted of untreated bacteria incubated under identical conditions. Each condition was tested in quadruplicate, and experiments were independently repeated three times.

### Inhibitory effects of mastic on hydrogen sulfide and methyl mercaptan production by P. gulae

Similarly, *P. gulae* suspensions (1 × 10⁸ CFU/mL) prepared as described above were co-incubated with mastic (0.25%, 0.5%, and 1%) for 5 min, and the production of hydrogen sulfide and methyl mercaptan was quantified using a gas chromatography system (OralChroma, Nissha FIS, Inc., Tokyo, Japan). Negative controls consisted of untreated bacterial suspensions, and buffer-only blanks were included to account for background. The direct deodorizing properties of mastic were assessed using a methyl mercaptan standard (FUJIFILM Wako Pure Chemical Corporation, Osaka, Japan). Each condition was tested in quadruplicate, and experiments were independently repeated three times.

### Inhibitory effects of mastic on pro-inflammatory cytokine secretion by P. gulae- or lipopolysaccharide-induced murine and canine macrophage cell lines

J774.1 cell lines (mouse monocyte-macrophage) and DH82 cell lines (canine macrophage) were obtained from the American Type Culture Collection (Manassas, VA, USA). J774.1 cells were cultured in Roswell Park Memorial Institute (RPMI) 1640 medium (FUJIFILM Wako Pure Chemical Corporation) supplemented with 10% fetal calf serum (FCS; Sigma-Aldrich Co., LLC., Tokyo, Japan) and penicillin–streptomycin (FUJIFILM Wako Pure Chemical Corporation). DH82 cells were cultured in Eagle’s minimum essential medium (EMEM; FUJIFILM Wako Pure Chemical Corporation) supplemented with 10% FCS and penicillin–streptomycin. Both cell lines (1 × 10^4^ cells/100 μL) were exposed to mastic (0.13%, 0.25%, 0.5%, and 1%) and 1 × 10^4^ CFU/mL of *P. gulae* or 1 μg/mL of lipopolysaccharide (LPS, Sigma-Aldrich Co. LLC.) for 24 h. Cytokine concentrations in the supernatants of J774.1 and DH82 cells were quantified using DuoSet ELISA Development Systems (R&D Systems, Minneapolis, MN, USA): IL-1β (DY401), IL-6 (DY406), and TNF-α (DY410). Absorbance was measured at 450 nm with wavelength correction at 570 nm using a microplate reader. The cytotoxicity of the mastic used in this experiment was assessed using the Cytotoxicity LDH Assay Kit-WST (Dojindo Laboratories, Kumamoto, Japan) before conducting the cytokine release assay.

### Inhibitory effects of mastic on P. gulae-induced phosphorylation of mitogen-activated protein kinase (MAPK) in J774.1

The phosphorylation levels of MAPKs including p38 and NFκB in J774.1 cells were measured 1 h after treatment with mastic (0.25%, 0.5%, 1%) treatment and exposure to 1 × 10^4^ CFU/mL of *P. gulae* using western blot analysis. Total proteins (30 µg) extracted from the cells using the M-PER™ Mammalian Protein Extraction Reagent (Thermo Fisher Scientific, Inc., Kanagawa, Japan) were separated using SDS-PAGE and transferred to PVDF membranes via Trans-Blot Turbo Transfer System (Bio-Rad Laboratories, Inc.). Primary antibodies (anti-phospho-p38, anti-p38, anti-phospho-NFκB, anti-NFκB, and anti-β-actin; Cell Signaling Technology, Inc., Danvers, MA, USA) were used for protein detection. They were visualized using a secondary antibody and then detected by ImmunoStar® Zeta (FUJIFILM Wako Pure Chemical Corporation). The iBright imaging system (Thermo Fisher Scientific) detected and quantified protein bands.

### Daily oral mastic treatments in dogs and cats with P. gulae-positive PD

All animal experiments were approved by the Animal Care and Use Committee of Azabu University (Approval No. 200318-1). All procedures were performed in accordance with the ARRIVE guidelines (https://arriveguidelines.org) and relevant institutional, national, and international regulations. Permission was obtained from all pet owners before including their pets in the study. Study designs are shown in Fig. 5A (dogs) and 6A (cats). The study included 40 dogs (ages 2–16 years, see Supplemental Table 1) and 15 cats (age 3–16 years, see Supplemental Table 2) with moderate-to-severe PD and no dental care during or within one month before the initiation of the clinical trial. Sample sizes (dogs: n = 40; cats: n = 15) were determined based on preliminary studies and a power calculation to detect significant changes in halitosis and gingivitis with 80% power at α = 0.05, accounting for potential variability in owner-administered gel application^19,20,35^. PD severity was classified by a licensed veterinarian based on AAHA dental care guidelines, using clinical signs of gingival inflammation and plaque accumulation. Scoring was performed by a single examiner blinded to treatment allocation. The dogs and cats were divided into two groups: daily mastic (5%)-containing gel treatment group (30 dogs and 10 cats) and daily vehicle control gel treatment group (10 dogs and 5 cats). Animals were randomly assigned to treatment or control groups using a computer-generated schedule, and all evaluators assessing gingivitis, plaque, and halitosis were blinded to group allocation. Pet owners were instructed to apply the gel daily but were not involved in outcome scoring. Dental gel was applied using the pet owner’s forefinger (no toothbrush or applicator was used) once daily for 30 min after the evening meal for 30–40 days. Owners were trained before the trial, and compliance was recorded in daily logs. Plaque and gingivitis were visually evaluated by a licensed veterinarian under standardized lighting conditions, based on the most severely affected teeth, using a 0–3 scale (0 = normal, 3 = most severe), without anesthesia or radiographs. No disclosing solution was used to avoid altering oral microbiota during the trial. The 30–40 day evaluation window was chosen to accommodate variation in owner compliance and the scheduling of follow-up visits, while still representing approximately one month of treatment. Gingivitis was scored according to redness, and swelling as follows: 0 = normal gingiva with no inflammation; 1 = mild gingival redness and swelling; 2 = moderate gingival redness and swelling; 3 = severe inflammation with marked redness and swelling. Plaque accumulation was scored as follows: 0 = no visible plaque; 1 = thin plaque deposit on tooth surface, detectable only after disclosing; 2 = moderate accumulation of soft deposits visible without disclosing; 3 = heavy plaque covering more than one-third of the tooth surface. A sensory evaluation was performed using questionnaires completed by pet owners after 30 days of treatment. Owners were asked to score their perception of halitosis and overall oral condition of their pets on a 5-point Likert scale (1 = very poor, 5 = very good). Hydrogen sulfide and methyl mercaptan levels in expired breath were measured using a gas chromatography system (Oral Chroma). *P. gulae* was detected in oral swab specimens collected from the gingival and/or subgingival margin and sulcus of the maxillary right or left canine and fourth premolar using a previously described polymerase chain reaction-based method^19^. Genomic DNA was extracted from each sample using the ISOFECAL kit (NIPPON GENE CO., LTD., Tokyo, Japan) and used as a template for two PCR assays: (i) a broad-range PCR targeting the 16S rRNA gene with primers 8UA and 1540R, and (ii) a targeted PCR using *P. gulae*-specific primers (forward: 5′-TTG CTT GGT TGC ATG ATC GG; reverse: 5′-GCT TAT TCT TAC GGT ACA TTC ACA). Reactions were performed in a total volume of 20 μL containing 2 μL of template DNA (20 μg/mL), Ex Taq DNA Polymerase (Takara Bio, Inc., Otsu, Japan), and primers, according to the manufacturer’s instructions. PCR amplification was carried out on a TaKaRa PCR Thermal Cycler Dice® Touch (Takara Bio, Inc.) using the following conditions for *P. gulae*-specific primers: initial denaturation at 95 °C for 4 min; 30 cycles of denaturation at 94 °C for 30 s, annealing at 62 °C for 30 s, and extension at 72 °C for 30 s; followed by a final extension at 72 °C for 7 min. Amplicons were separated on 1% agarose using the Invitrogen™ E-Gel™ Power Snap system (Thermo Fisher Scientific Inc.) and visualized with SYBR staining. The enzymatic activity hydrolyzing N-benzoyl-DL-arginine-naphthylamide (BANA), a marker for severe periodontal pathogens, including *Treponema denticola*, *P. gingivalis, P. gulae,* and *Bacteroides forsythus,* was assessed using the BANA test (BANAMet LLC, MI, USA)^36^.

### Statistical analysis

All data are expressed as the mean ± standard error of the mean (SEM). To evaluate multigroup experiments for the in vitro study, we performed an analysis of variance (ANOVA), followed by Dunnett’s multiple comparison test. For the clinical research, we performed two-way ANOVA, followed by the uncorrected Fisher’s least significant difference test. Statistical significance was estimated at a 5% probability level. Data were analyzed using GraphPad Prism 10 (GraphPad Software, San Diego, CA, USA).

## Humans and animal rights

All experimental protocols were approved by the Animal Care and Use Program of Azabu University (Approval No. 200318–1). All methods are reported in accordance with ARRIVE guidelines (https://arriveguidelines.org), relevant guidelines and regulations. Permission was obtained from all pet owners before including their pets in the study.

## Supplementary Information

Below is the link to the electronic supplementary material.

Supplementary Material 1

Supplementary Material 2

Supplementary Material 3

## Data Availability

The data are available from the corresponding author upon reasonable request.
