# Supplementary figures and images for: Whole mastic resin ameliorates halitosis and gingivitis in dogs and cats infected with Porphyromonas gulae
Source: Sci Rep. 2025 Dec 8;15:43332. doi: 10.1038/s41598-025-27244-x (PMC12685946; doi:10.1038/s41598-025-27244-x)

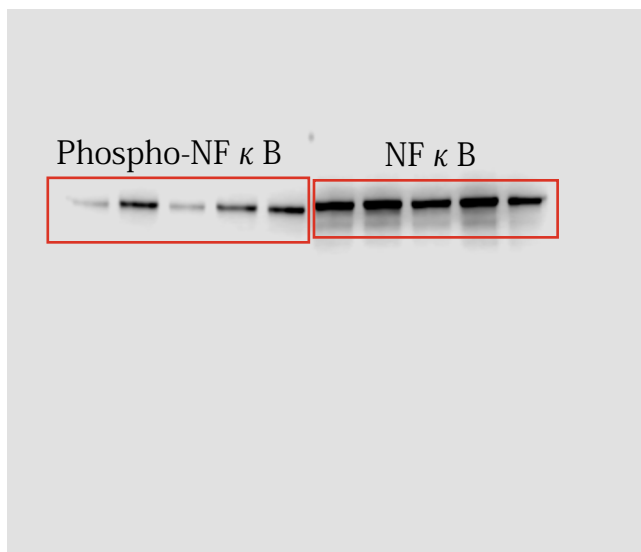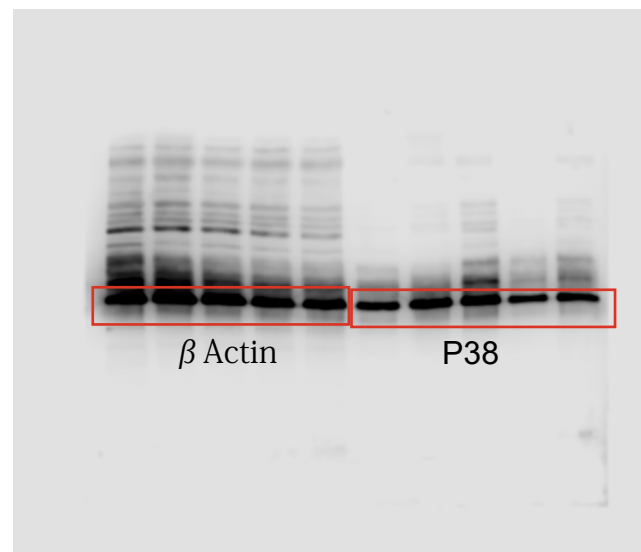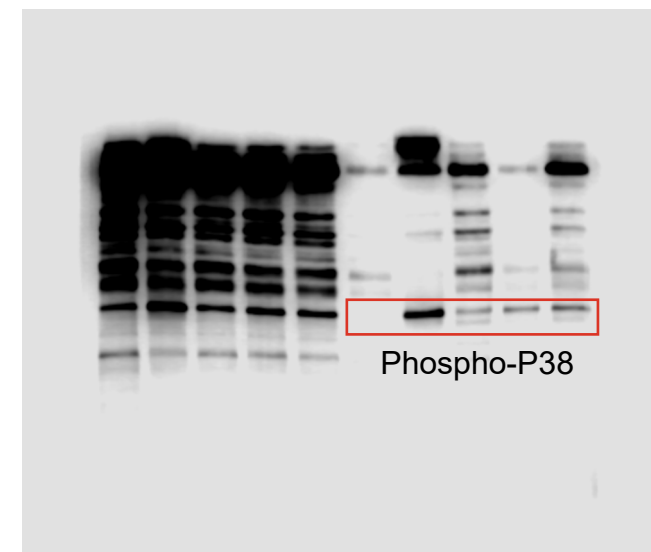

Supplement: Supplementary file 1 — Supplementary Material 1 [file 41598_2025_27244_MOESM1_ESM.pdf]
