# Supplementary material for: Whole mastic resin ameliorates halitosis and gingivitis in dogs and cats infected with Porphyromonas gulae
Source: Sci Rep. 2025 Dec 8;15:43332. doi: 10.1038/s41598-025-27244-x (PMC12685946; doi:10.1038/s41598-025-27244-x)
Supplement: Supplementary file 2 — Supplementary Material 2 [file 41598_2025_27244_MOESM2_ESM.docx]

**Supplemental Figure. 1** The direct deodorant properties of the mastic were not observed in the methyl mercaptan standard. Each result is presented as the mean (ppb) ± 1 SEM. n = 4 per group.

**Supplemental Figure. 2** Pet owners’ sensory evaluation of halitosis showed significant improvement following mastic treatment for 30-40 days compared to pre-treatment and vehicle control values in dogs. Each result is presented as mean ± 1 SEM. n = 30 in the mastic treatment group and n = 10 in the vehicle control group. P < 0.05 (uncorrected Fisher's least significant difference test).

**Supplemental Figure. 3** Pet owners’ sensory evaluation of halitosis showed significant improvement following mastic treatment for 30-40 days compared to pre-treatment and vehicle control values in cats. Each result is presented as mean ± 1 SEM. n = 10 and n = 5 for the mastic treatment and vehicle control groups, respectively. P < 0.05 (uncorrected Fisher's least significant difference test).

**Supplemental Figure. 1**

**Supplemental Figure. 2**

**Supplemental Figure. 3**
