# Supplementary material for: Whole mastic resin ameliorates halitosis and gingivitis in dogs and cats infected with Porphyromonas gulae
Source: Sci Rep. 2025 Dec 8;15:43332. doi: 10.1038/s41598-025-27244-x (PMC12685946; doi:10.1038/s41598-025-27244-x)
Supplement: Supplementary file 3 — Supplementary Material 3 [file 41598_2025_27244_MOESM3_ESM.docx]

**Supplemental TABLE 1**

Dog breeds included in the clinical study.

| Group | Breeds | Age | Sex |
| --- | --- | --- | --- |
| Mastic treated group  (n=30) | Yorkshire terrier | 11 years | Female (spay) |
|  | Toy Poodle | 5 years | Male (castration) |
|  | Miniature Dachshund | 16 years | Female (spay) |
|  | Maltese × Toy Poodle | 3 years | Male (castration) |
|  | Toy Poodle | unknown | Female (spay) |
|  | Maltese × Yorkshire terrier | 5 years | Male (castration) |
|  | Toy Poodle | 11 years | Female (spay) |
|  | Shiba | 4 years | Female (spay) |
|  | Labrador Retriever | 6 years | Female (spay) |
|  | Toy Poodle | 9 years | Male (castration) |
|  | Toy Poodle | 7 years | Male (castration) |
|  | Yorkshire terrier | 4 years | Female (spay) |
|  | Chihuahua | 6 years | Female (spay) |
|  | Miniature Dachshund | 9 years | Male (castration) |
|  | Miniature Dachshund | 6 years | Female (spay) |
|  | Maltese × Chihuahua | 2 years | Female (spay) |
|  | Miniature Dachshund | 7 years | Female (spay) |
|  | Miniature Dachshund | 8 years | Female (spay) |
|  | Toy Poodle | 8 years | Male (castration) |
|  | Toy Poodle | 6 years | Female (spay) |
|  | Chihuahua | 6 years | Female (spay) |
|  | Toy Poodle | 16 years | Male (castration) |
|  | Dachshund × Toy Poodle | 11 years | Female (spay) |
|  | Dachshund × Toy Poodle | 10 years | Female (spay) |
|  | Toy Poodle | 4 years | Female (spay) |
|  | Pomeranian × Spitz | 2 years | Male (castration) |
|  | Pomeranian | 5 years | Female (spay) |
|  | Italian Greyhound | 12 years | Female (spay) |
|  | Pomeranian | 11 years | Male (castration) |
|  | Jack Russell | 8 years | Male (castration) |
| Vehicle control group  (n=10) | Toy Poodle | 12 years | Male (castration) |
|  | Shiba | 9 years | Female (spay) |
|  | Toy Poodle | 14 years | Female (spay) |
|  | Miniature Dachshund | 6 years | Female (spay) |
|  | Chihuahua | 9 years | Female (spay) |
|  | Miniature Dachshund | 7 years | Female (spay) |
|  | Pomeranian | 7 years | Male (castration) |
|  | Papillon | 8 years | Female (spay) |
|  | Beagle | 9 years | Female (spay) |
|  | Italian Greyhound | 6 years | Male (castration) |

**Supplemental TABLE 2**

Cat breeds included in the clinical study.

| Group | Breeds | Age | Sex |
| --- | --- | --- | --- |
| Mastic treated group  (n=10) | Mix | 15 years | Female (spay) |
|  | Mix | 8 years | Male (castration) |
|  | Mix | 16 years | Male (castration) |
|  | Mix | 7 years | Male (castration) |
|  | Mix | 9 years | Female (spay) |
|  | American Shorthair | 3 years | Male (castration) |
|  | Mix | 8 years | Female (spay) |
|  | Mix | 11 years | Male (castration) |
|  | [Scottish](https://eow.alc.co.jp/search?q=Scottish&ref=awlj) [Fold](https://eow.alc.co.jp/search?q=Fold&ref=awlj) | 6 years | Female (spay) |
|  | Siberian | 4 years | Male (castration) |
| Vehicle control group  (n=5) | Mix | 8 years | Female (spay) |
|  | Mix | 15 years | Female (spay) |
|  | Mix | 4 years | Male (castration) |
|  | Mix | 3 years | Male (castration) |
|  | Mix | 9 years | Female (spay) |
